# Characterization of Spatter and Sublimation in Alloy 718 during Electron Beam Melting

**DOI:** 10.3390/ma14205953

**Published:** 2021-10-10

**Authors:** Ahmad Raza, Eduard Hryha

**Affiliations:** Department of Industrial and Materials Science, Chalmers University of Technology, SE-412 96 Gothenburg, Sweden; hryha@chalmers.se

**Keywords:** electron beam melting, spatter formation, sublimation, metalization, oxidation, metal powder, powder reuse

## Abstract

Due to elevated temperatures and high vacuum levels in electron beam melting (EBM), spatter formation and accumulation in the feedstock powder, and sublimation of alloying elements from the base feedstock powder can affect the feedstock powder’s reusability and change the alloy composition of fabricated parts. This study focused on the experimental and thermodynamic analysis of spatter particles generated in EBM, and analyzed sublimating alloying elements from Alloy 718 during EBM. Heat shields obtained after processing Alloy 718 in an Arcam A2X plus machine were analyzed to evaluate the spatters and metal condensate. Comprehensive morphological, microstructural, and chemical analyses were performed using scanning electron microscopy (SEM), focused ion beam (FIB), and energy dispersive spectroscopy (EDS). The morphological analysis showed that the area coverage of heat shields by spatter increased from top (<1%) to bottom (>25%), indicating that the spatter particles had projectile trajectories. Similarly, the metal condensate had a higher thickness of ~50 μm toward the bottom of the heat shield, indicating more significant condensation of metal vapors at the bottom. Microstructural analysis of spatters highlighted that the surfaces of spatter particles sampled from the heat shields were also covered with condensate, and the thickness of the deposited condensate depended on the time of landing of spatter particles on the heat shield during the build. The chemical analysis showed that the spatter particles had 17-fold higher oxygen content than virgin powder used in the build. Analysis of the metalized layer indicated that it was formed by oxidized metal condensate and was significantly enriched with Cr due to its higher vapor pressure under EBM conditions.

## 1. Introduction

Electron beam melting (EBM) is an effective additive manufacturing (AM) technique due to its capacity to produce multilayers of dense and geometrically complex parts in a single build with low residual stresses and minimal support structures [1]. For better energy efficiency and to circumvent the smoking effect, a high vacuum in the chamber to minimize electron beam attenuation and preheating of the powder bed to semi-sintering temperature to enhance the electronic conductivity of the powder bed are prerequisites of EBM [2,3]. Although preheating at elevated temperatures and high vacuum can improve the process stability, these processing conditions can affect the unused feedstock powder in the build chamber [4,5,6].

Preheating at elevated temperatures can result in surface oxidation of the powder bed, a prevalent powder degradation mechanism in EBM [7,8]. The preheating temperature varies and depends on the sintering capability of each alloy. For example, the preheating temperature for Alloy 718 is ~1000 °C, and that for TiAl6V4 is ~650 °C [9,10]. Hence, elements with high susceptibility to oxidation, such as Al, Cr, and Ti, can quickly oxidize at such extreme temperatures, even under a high vacuum, if the oxygen potential is above equilibrium [11,12]. A study on Alloy 718 by Gruber et al. [4] showed severe surface oxidation of reused powder after a few cycles. Aluminum (Al) was the main oxide-forming element and formed Al-based oxide particulates. Due to the particulate nature of oxides and the lack of uniform passive layer formation, further reuse of powder increased the amount of Al-based oxide particulates. A follow-up study related to the role of powder oxidation in defect formation showed the prevalence of oxide inclusion in built samples [6], which is potentially detrimental to the properties of the final product. Moreover, the oxidation mechanism is relatively material-dependent; that is, the oxidation mechanism in TiAl6V4 differs slightly from that in Alloy 718, where higher oxygen solubility determines the extent of powder degradation [13,14]. After reusing TiAl6V4 powder for 30 cycles under EBM conditions, Montelione et al. demonstrated an increased oxygen content after each cycle [15]. Similarly, Ghods and coworkers [11] showed an increase in oxygen content from 0.14% in virgin powder to 0.20% in reused after 11 cycles. A follow-up study showed the effect of powder reuse on porosity formation during the processing of TiAl6V4, where no distinguishable change in porosity in fabricated parts was observed even after 30 cycles of powder reuse [16]. However, the effect of oxygen enrichment in Ti alloys on mechanical properties is well known where an increase in oxygen content increases the hardness and decreases the room temperature ductility [17,18]. Such a study on EBM parts was conducted by Po-pov et al. [19] on TiAl6V4, where the elongation and breakage area decreased in parts fabricated through reused powder.

Additionally, the combined effect of high vacuum and preheating can cause sublimation of metallic elements and further condensation of metal vapors on the surrounding build area surfaces, such as the heat shields, known as metalization [20,21]. The metalization behavior of Al and its effect in TiAl was documented in an earlier study by Cormier et al. [22], who showed an Al content decrease of up to 7 at% in the fabricated part compared to the original powder. Similarly, in a study by Petrovic et al. [12] on TiAl6V4, a 3% decrease in Al content was observed in reused powder after 12 cycles. Additionally, a study on compositionally complex Al0.5CrMoNbTa0.5 high entropy alloy showed a decrease in the content of Al to 8.4 at% in build sample as compared to 12 at% in mechanically alloyed powder [23]. Some studies have recently proposed metalization control mechanisms, including vacuum [24] and process parameter control [25]. Damri et al. [24] suggested increasing the pressure in the build chamber by one order of magnitude (from 10^−3^ to 10^−2^ mbar). This did not significantly affect the energy efficiency of the electron beam but could help reduce the evaporation of elements. Regarding process parameters, Schwerdtfeger et al. [25] showed that the evaporation of Al could be reduced to 0.5 at% in the fabricated product compared to the original powder by reducing the beam energy and layer thickness.

Alloy 718 is one of the most interesting alloys processed by EBM due to its excellent high-temperature properties and good processability with EBM [10]. However, knowledge is lacking regarding spatter formation and its characteristics, as well as sublimation/metalization during the processing of Alloy 718. Alloy 718 includes Al and Cr, elements with high vapor pressures at elevated temperatures, which are critical for achieving superior properties in the final product, making it important to understand the sublimation of these elements during EBM. Another unclear aspect of EBM is the spatter formation mechanism. Spatter generation is a well-documented phenomenon in laser powder bed fusion process (LPBF), due to the potential for collecting spatter powder from the gas inlet and outlet [26,27,28,29]. In EBM, it is impossible to retrieve and investigate the spatters from sintered powder cake. Thus, the development of a methodology allowing robust sampling and analysis of the spatter is required.

In the current study, heat shields were investigated to evaluate the spatters and sublimation/metalization. The careful sampling and high-resolution analysis enabled the determination of the nature of spatters and condensate on the heat shield. Detailed chemical analysis revealed the predominant sublimating and oxidizing elements during EBM.

## 2. Materials and Methods

The material processed for this study was virgin commercially pre-alloyed 718 powder produced by plasma atomization of wire in an argon atmosphere. The composition of Alloy 718 used in this study is given in Table 1, with a particle size distribution of 45 to 105 μm.

**Figure 1:**
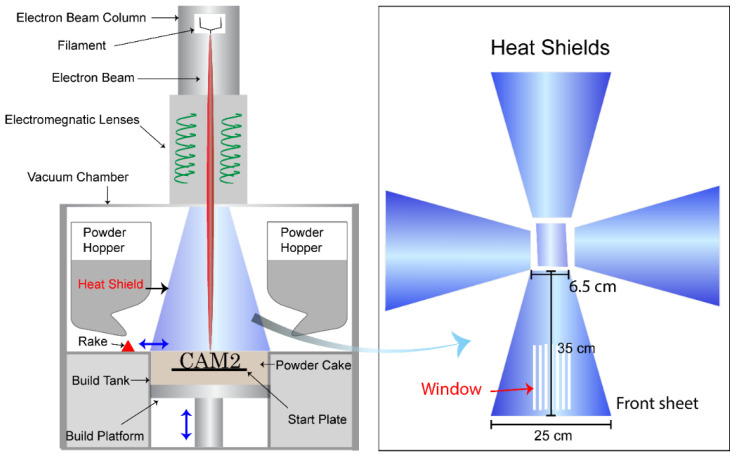
Schematic of EBM machine with an illustration of the heat shields.

An Arcam EBMA2X machine, located at GE Additive, Gothenburg, Sweden, was used to process the powder and obtain the heat shields. New heat shields were used for the purpose of this experiment. Heat shields are used in EBM machines to protect the surrounding equipment from the effects of temperature and vapor condensation, (Figure 1). Heat shields are usually made from 316L stainless steel sheets. The sheets used in the current work had a height of 35 cm and were used for 100 h of build time while processing Alloy 718 powder. An ultra-high vacuum of ~10^−5^ mbar was achieved in the chamber before starting the electron beam to remove the residual oxygen from the chamber, in order to avoid powder oxidation. While processing, He gas (grade 5) was introduced, increasing the pressure to ~10^−3^ mbar. The powder bed temperature was maintained at 1000 °C to pre-sinter the powder cake to enhance the conductivity and avoid the smoking effect. After a build of 100 h machine time, all four heat shields (as schematically shown in Figure 1) were carefully removed to avoid contamination of the sheets. These sheets were further used to collect the spatters and condensate for characterization.

The sheet was cut into small samples, and high-resolution scanning electron microscopy (HR-SEM) analysis was performed using a LEO Gemini 1550 SEM (ZEISS, Oberkochen, Germany) equipped with an INCA X-sight detector for energy dispersive spectroscopy (EDS) to assess the morphology of the spatter powder and condensate deposited on the heat shields. For cross-sectional analysis of the deposited spatter particles, focused ion beam (FIB) etching was performed using FEI Versa 3D FIB equipment. FIB cross-sectioning is a convenient way to observe the microstructure of powder particles. In the current study, FIB was helpful to monitor the directional variation in the microstructure of spatters deposited on the heat shield. S It was not possible to obtain such information in metallographically prepared spatter particle cross-sections. For further analysis, spatter particles were removed from the sheet and mounted in conductive epoxy; metallography was then performed. Electrolytic etching using oxalic acid was conducted to reveal the condensate deposited on the particle surfaces.

A comparison of the bulk oxygen contents of collected spatters with virgin Alloy 718 powder was performed by a combustion technique using a LECO ON836 analyzer. X-ray photoelectron spectroscopy (XPS) using a PHI 5500 (Physical Electronics, Chanhassen, MN, USA) instrument was conducted to further confirm the elements present in the condensate and at the spatter particle surfaces. The PHI 5500 instrument was equipped with a monochromator Al Kα source (1486.6 eV). The powder particles were mounted on a pure indium plate by light pressing to avoid damage to the powder surface during sample preparation.

## 3. Results

**Figure 2:**
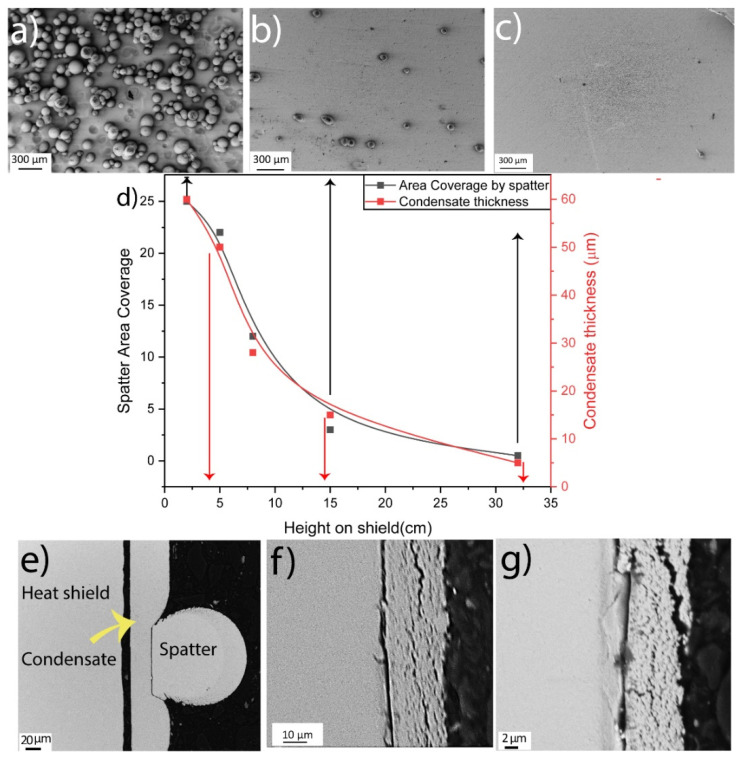
The top micrographs show area coverage by spatters from the (**a**) bottom (2 cm), (**b**) middle (15 cm), and (**c**) top (~32 cm) of the heat shield (distances from the bottom of the heat shield). The center plot (**d**) shows the variation in area coverage by spatter particles and condensate thickness with height on the heat shield. The bottom micrographs show the condensate thickness (**e**) at the bottom (2 cm), (**f**) middle (15 cm), and (**g**) top (32 cm) of the heat shield.

SEM analysis of heat shields showed a variation in the distribution of spatter particles and deposited metal condensate. Figure 2 shows the relationships of spatter area coverage and condensate thickness with the height on the heat shield. The distributions of spatter particles at 2, 15, and 32 cm height on the heat shield, (Figure 2a–c) differ regarding area coverage by spatters on the heat shield, where spatters cover 25% of the surface at the bottom. The area coverage drastically decreased to around 5% at 15 cm height and ~1% at 32 cm height. This suggests that the motion of spatter particles is projectile, where most of the spatters land on the powder cake.

The condensate thickness also exhibited a similar tendency, with a thickness of >50 μm at the bottom of the heat shield, which decreased to around 15 μm at 15 cm height and less than 5 μm at 32 cm height, (Figure 2e–g). The micrographs shown in Figure 2e clearly show that spatter deposition and metal sublimation/condensation on the heat shield are continuous processes, occurring over the whole build process. As shown in Figure 2e, a layer of condensate is continuously deposited. Midway through the EBM, spatter particles landing on the condensate on the heat shield continued to grow on the heat shield, and on already-deposited spatter particles. This was confirmed by evaluating the thickness of the condensate layer, which was approximately equal to the sum of the condensate thickness below the spatter particles and the layer deposited on the spatter particles.

**Figure 3:**
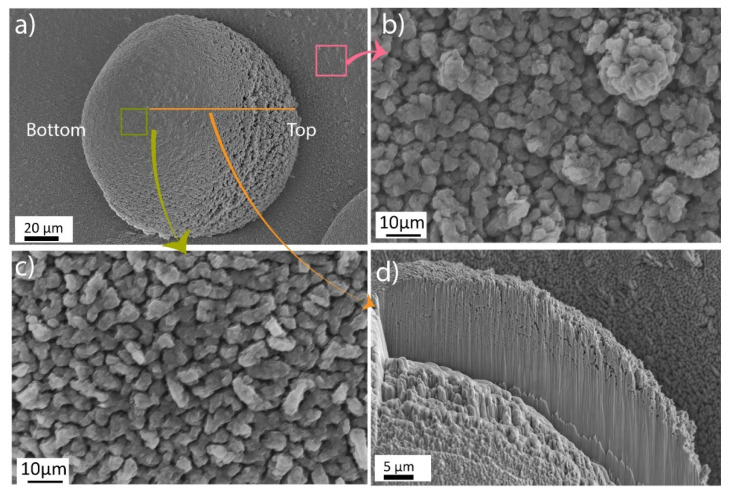
Morphological analysis of (**a**) spatter particle deposited on the heat shield surface and high-magnification micrographs of (**b**) condensate on the surface shield, (**c**) spatter particle surface, and (**d**) FIB cross-section of spatter particle.

The morphology of the spatter and condensate deposited on the heat shield is shown in Figure 3. The spatter particle has a teardrop shape with slight elongation toward the bottom, (Figure 3a). The teardrop shape of the spatter indicates inhomogeneous metal condensation on the spatter surface after its deposition. This is also confirmed by the surface appearance of the spatter particle, which has a dense morphology toward the bottom and a porous morphology toward the top, (Figure 3). A comparison between the dense part of the particle surface, (Figure 3c) and the condensate deposited on the heat shield, (Figure 3b) highlights the similarity in morphology, indicating that the surface of the spatter, sampled from the heat shield, is covered with condensate of vapors originating from the melt pool. A focused ion beam (FIB) cross-section of a spatter particle is presented in Figure 3d to evaluate the preferential direction of vapor deposition on the spatter surface. Careful analysis of the morphology and porosity in the cross-section indicated variation in the amount of condensate. Interestingly, the bottom of the spatter particle, which had a porous surface morphology, was dense. The porosity increased toward the top of the particle due to the higher thickness of the condensate, which was porous. Thus, the similar morphologies of the condensate layer and spatter particles deposited on the heat shield confirm the presence of the condensate on the spatter particle surfaces, (Figure 3b,c). This also indicates that the vapors undergo bottom-up deposition on the heat shield, which is also evident in Figure 2c. However, an important conclusion from this observation is that spatter particles sampled from the heat shield are not representative of the spatter particles produced during EBM as they are covered with a significant layer of the oxidized metal condensate; that is, condensate on the spatter particle surfaces after their deposition on the heat shield.

**Figure 4:**
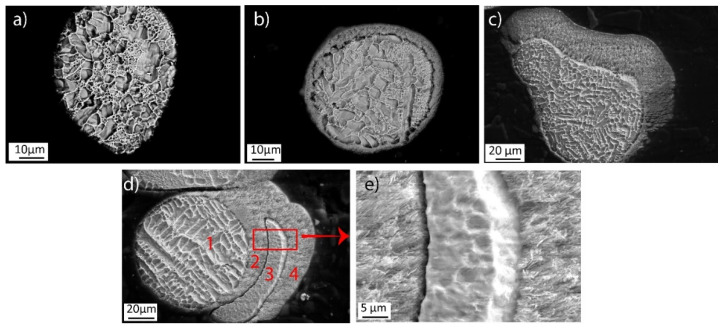
A comparison of condensate deposited on the surface of spatters: (**a**) spatter particle without condensate, (**b**) spatter particle with a condensate of approximately 5–10 μm thickness, (**c**) spatter particle with a condensate thickness of approximately 40 μm, (**d**) multiple layers of condensate and spatter, and (**e**) high-magnification inset from (**d**).

Microstructural analysis of an etched cross-section of spatter particles sampled from the heat shields was conducted to further evaluate the spatter particles and the evolution of condensation on their surface. The micrographs shown in Figure 4 are useful in elucidating the sequence of events occurring during EBM. The spatter particle in Figure 4a has a clean surface with no evidence of condensation, suggesting that it landed on the heat shield when the build cycle was about to finish, and insufficient time was available for the metal to condense on the surface. However, the particle shown in Figure 4b probably landed after the EBM process was half-finished and, hence, had a condensate layer of around 5–10 μm on the surface. Similarly, the particle shown in Figure 4c possibly was deposited at the start of the build and had a thick condensate layer around it. The thickness of the condensate layer is about 40 μm, comparable to the thickness of the condensate observed at the bottom of the heat shield shown in Figure 2c. Figure 4d shows an interesting particle presenting a sequence of four events that further clarify the spatter deposition and condensation on the heat shield. During the first stage, an ejected particle from the melt pool was deposited on the heat shield. As EBM continued, metal evaporation and its further condensation on the heat shields and deposited spatter particles continued. When the condensate thickness reached ~20 μm (about half the process time), another molten particle was deposited on the condensate layer. This was again followed by metal condensation until the end of EBM. Again, this confirms that the metalization from the melt pool and condensation at the heat shields is a continuous process. The spatter particles on the heat shield surface can have varying thicknesses of surface condensate depending on the at which time these particles landed on the heat shield during the build. The etched cross-section also reveals that the condensate has a hairy needle-like structure, typical for vapor deposition, and is loosely attached to the particle surface, (Figure 4e).

**Figure 5:**
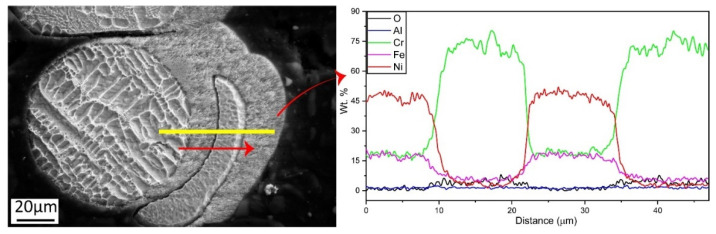
EDS line scan on highlighted yellow line in SEM micrograph.

Based on the difference between the microstructures of spatter and condensate, an EDS line scan was performed on the particle shown in Figure 4d to investigate the change in chemistry. Figure 5 shows the result of the EDS line scan, illustrating four distinct regions. The spatter particles in the first and third areas have a nominal composition corresponding to Alloy 718. However, the condensate composition in the second and fourth areas is dominated by Cr with minute amounts of Ni and Fe. An increase in oxygen in the condensate is also observed. This indicates that Cr is the predominantly evaporating element in Alloy 718. It also reacts with oxygen and deposits as Cr-based oxide on the heat shield surface.

**Figure 6:**
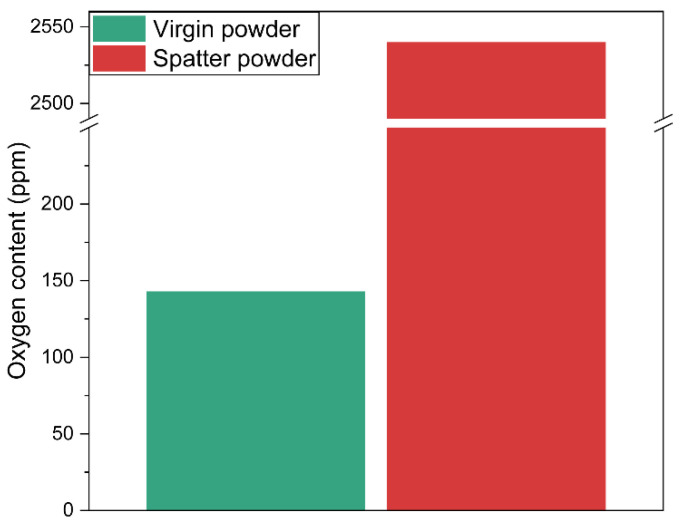
A comparison of oxygen contents of virgin powder and spatter collected from heat shield.

Considering the high oxygen content registered in the condensate by EDS analysis, (Figure 5), the oxygen content in the accumulated spatter was measured by combustion analysis and compared to the virgin feedstock powder, (Figure 6). The oxygen content of virgin powder used during the build was <150 ppm. However, a drastic surge in the oxygen content was observed in spatter particles collected from the heat shield up to a value of 2540 ppm. This is a ~17-fold increase compared to the virgin powder, whereas in the previous study [1,2], an oxygen increase of only up to 30% was registered in the reused powder after one run and linked to the high-temperature oxidation of the feedstock powder due to the high temperature of the build (around 1000 °C) and prolonged exposure (100 h in this case) at the oxygen level below equilibrium [30]. Considering the extremely high oxygen content, if these spatters exfoliate from the heat shield and fall on the powder bed, they can create detrimental oxide-based defects in EBM products. However, neither such an oxidized powder particle [1] nor such a large oxide inclusion [2] was observed when processing the same material in a similar setup by the authors for the same number of cycles, assuming that the probability of such exfoliation is moderately low (if heat shields are changed periodically). Thus, the risk of build contamination by such a spatter with the oxidized condensate is minimal for the material and setup used.

**Figure 7:**
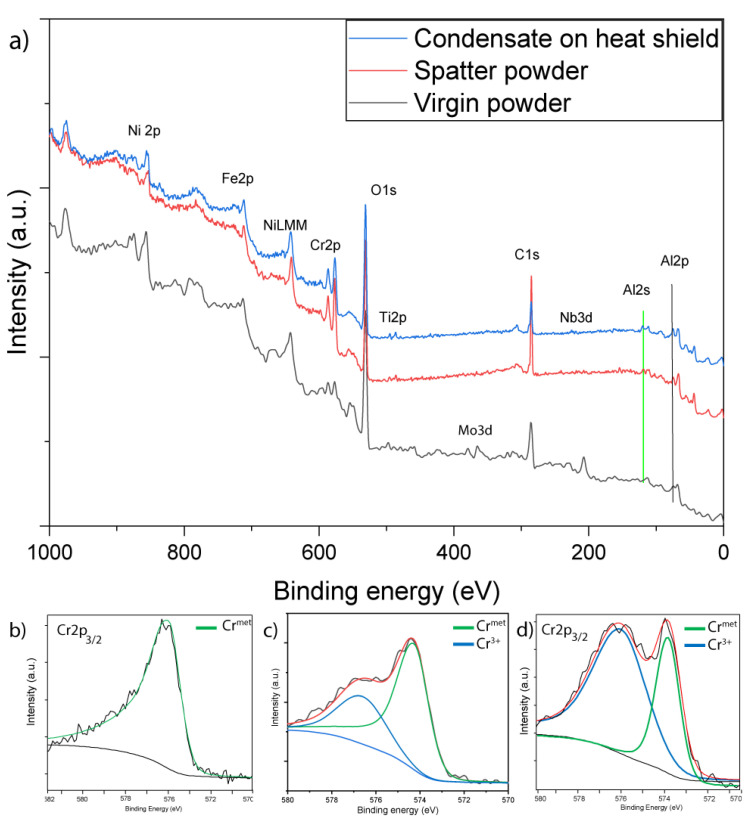
A comparison of XPS (**a**) survey spectra of virgin powder with spatters and condensate deposited on heat shield along with fitted narrow scans of (**b**) virgin powder, (**c**) spatter particles, and (**d**) condensate at 30 nm etch depth.

XPS analysis can provide useful information regarding chemical changes in the elements present in condensate compared to the virgin powder. Figure 7a compares XPS survey spectra of virgin powder, spatter particles, and condensate on a heat shield. The peaks of oxygen (O1s), carbon (C1s), and nickel (Ni2p) are distinguishable, along with peaks of iron (Fe2p), chromium (Cr2p), titanium (Ti2p), niobium (Nb3d), and aluminum (Al2p). The survey spectra for spatter powder and condensate were almost identical, reaffirming the aforementioned results where the surfaces of spatter particles were covered with condensate. However, these spectra were significantly different from those of virgin powder, where more significant Cr and O peaks could be observed for the spatter and condensate. However, clear peaks of alloying elements such as Nb3d and Mo3d could be observed for the virgin powder but not for the spatter and condensate. The relative intensity of Cr2p was substantially higher in spectra from the spatter powder and condensate, and peaks for other oxide-forming elements such as Al2p and Ti2p were also more pronounced for the spatter and condensate. These findings highlight that the condensate and spatter particles were highly oxidized compared to the virgin powder.

A comparison of fitted Cr2p_3/2_ narrow scans from virgin powder, spatter powder, and condensate at 30 nm etch depth is presented in Figure 7b–d to further analyze the oxidation level. The fitted spectra from virgin powder, (Figure 7b) showed complete removal of the surface oxide and the presence of only metal at this etch depth, confirming the presence of only a thin layer of oxide in the virgin powder [1]. Conversely, the fitted spectra for spatter particles and condensate showed 33% and 70% Cr in oxide form, respectively, even at this etch depth. This could be due to the partial oxidation of metal vapor during or just after the deposition by the oxygen present in the vacuum chamber. Room-temperature oxidation of the metalized vapor after the process is finished and the process chamber is cooled and exposed to air will result in a significantly thinner oxide passivation layer. Another contribution could be from the sublimation of Cr oxide from the powder surface during the interaction between the electron beam and powder, as Cr is present in the oxide state even on the surface of the virgin powder, but in significantly lower quantities [1].

**Figure 8:**
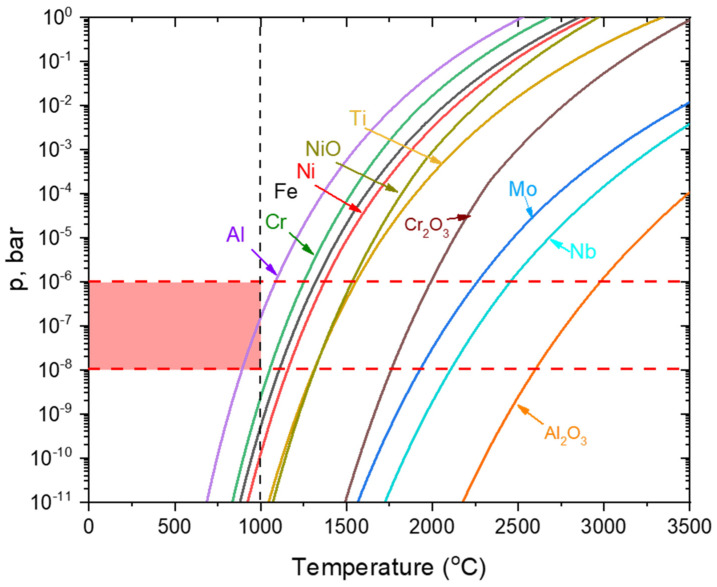
Sublimation pressures of metallic elements present in Alloy 718, based on HSC Chemistry 9.7 data.

However, as shown in Figure 8, the sublimation pressures of the oxides for the elements of interest (Ni, Cr, and Al) are significantly lower than those for the pure metals, assuming that metal vapors were oxidized during/directly after the deposition and not sublimated as oxides. Additionally, it is noteworthy that Al has the highest equilibrium sublimation pressure from the alloying elements present, followed by Cr. However, due to its much lower amount in the alloy (~0.5 wt.% compared to ~20 wt.% of Cr), the observed Al content in the condensate is much lower than that of Cr, which forms the base of the metal condensate based on the EDS, (Figure 5) and XPS, (Figure 7) analyses showing that the Ni content was almost an order of magnitude lower than the Cr content. This is consistent with the equilibrium partial pressure diagram, (Figure 8), where the sublimation pressure of Al is close to the pressure in the vacuum chamber during EBM, followed by Cr, resulting in significant sublimation and depletion of these elements during EBM. The effects of this Al and Cr sublimation on the properties of the components (e.g., amount of the γ’ and high-temperature and corrosion properties) when reusing feedstock powder should be further evaluated.

## 4. Conclusions

Spatters and metal condensate gathered from heat shields from EBM of Alloy 718 were characterized. Based on the HR-SEM, XPS, chemical analyses, and thermodynamic evaluation of the metal sublimation for spatter and condensate from EBM of Alloy 718, the following conclusions were drawn:

- Significant sublimation of Cr and Al during EBM of Alloy 718 was detected. Metal condensate is deposited on the heat shields and further oxidized during/directly after deposition. Metal vapors condense close to the build area, resulting in the thickness of the metalized layer reaching 50 μm at the bottom of the heat shield and decreasing to less than 5 μm at ~32 cm height.
- Spatter particles are also deposited on the heat shields close to the build area, with about 25% coverage at the bottom of the heat shield and less than 1% coverage at the top. Spatter particles are also covered by the oxidized metal condensate, the thickness of which is determined by the time at which the spatter particles are located on the heat shield. This resulted in a significant increase in the oxygen content of such a spatter with the metal condensate, reaching approximately 2540 ppm, about 17-times higher than with virgin powder.
- Spatter particles on heat shields are covered with oxidized metal condensate; hence, they are not representative of the spatter created during EBM. Simultaneously, spatter particles on heat shields have excellent cohesion to the heat shields; hence, they are not expected to pose a significant risk of material contamination during EBM if heat shields are properly maintained and periodically changed.
- Cr predominantly forms metal condensate with traces of Ni and Al. This is consistent with the equilibrium partial pressure of metallic elements under the conditions used during EBM of Alloy 718. Although Al has about two orders of magnitude higher sublimation pressure than Cr, its much lower content in Alloy 718 compared to Cr results in more significant sublimation of Cr. The effects of Al and Cr loss on material properties when reusing feedstock powder should be further studied to evaluate the impact of sublimation of alloying elements during EBM of Alloy 718.

## Figures and Tables

**Table 1:** Chemical composition of the Alloy 718 powder as provided by the powder producer.

Elements	Ni	Co	Cr	Mo	Ti	Mn	Nb	Ta	Al	Fe	Si	C
wt.%	54.1	0.04	19.0	2.99	1.02	0.12	4.97	<0.01	0.52	17.12	0.06	0.03
at%	53.38	0.04	21.16	1.80	1.23	0.13	3.10	<0.01	1.12	17.75	0.12	0.14

## Data Availability

Not applicable.
